# The Role of Common Ground on Object Use in Shaping the Function of Infants’ Social Gaze

**DOI:** 10.3389/fpsyg.2020.00619

**Published:** 2020-04-16

**Authors:** Nevena Dimitrova

**Affiliations:** Haute École de travail social et de la santé (HETSL), Haute École Spécialisée de Suisse Occidentale (HES-SO), Lausanne, Switzerland

**Keywords:** eye gaze, communicative function, infancy, parent–child interaction, socio-materiality, object use, early development

## Abstract

Although infants’ social gaze has specific communicative functions, it remains unclear what they are. In this conceptual analysis paper, we provide a theoretical framework for the study of the functional aspects of eye gaze in early childhood. We argue that studying the communicative functions of infants’ eye gaze involves three premises: the centrality of the object, the importance of common ground on object use, and the role of parental interpretations. The ability to communicate intentionally begins when infants start referring to external objects. Beyond dyadic – infant–parent – emotional sharing, infant social gaze within the infant–parent–object triad becomes an increasingly complex communicative modality. As the predominant type of communicative referents in infancy, objects are thus central to early communication. Although they have affordances, objects are used in conventional ways shared between users (i.e., common ground). Parents transmit to infants the socio-cultural use of objects, which infants progressively learn and master. Accordingly, we argue that within early triadic interactions, the communicative function of infants’ eye gaze is shaped by the knowledge that the infant and the parent share on the socio-cultural use of the referent (i.e., the object). Importantly, before young children develop their ability to convey clear communicative functions, including with eye gaze, the interpretations and responses that parents provide to infants’ early communicative acts play a major role. Relying on these premises, we argue that when referring to objects for which the infant and the parent share common ground, the function of the infant’s social gaze becomes communicatively meaningful for the parent. The knowledge on the communicative referent (i.e., the object) shared between the infant and the parent thus shapes the course of communicative behavior, constitutes and reflects the interactive function of gaze, and cues parents into tailoring their communicative response according to the infant’s developmental needs. Through this theoretical framework for the study of the communicative function of infant eye gaze, an emphasis is put on the key role that socio-materiality plays in early communicative development.

## Introduction

As social beings, infants seek social interaction from the very beginning of life. They produce a myriad of communicative behaviors – first without intention, then intentionally – in order to communicate with their social partners (Rochat, 2001). Infants do so in order to express their internal state, to share emotional experiences, and later on, to influence their communicative partners. Among the earliest communicative means infants use is eye gaze (Volkmar and Mayes, 1990). Progressively, infants come to produce other (smiles, cries, vocalizations) and more complex (postures, gestures) communicative means (Adamson, 1996); however, eye gaze remains a principal component of infants’ earliest communicative acts. While the question of communicative intentionality in infancy has been the subject of a longsome debate, there are different established criteria allowing characterization of an infant’s communicative bid as intentional. Importantly, a key criterion for the earliest manifestations of intentional communication is that an infant’s eye gaze is oriented toward the communicative partner (Bruner, 1973; Bates, 1979; Franco and Butterworth, 1996). Despite the centrality of an infant’s eye gaze in early communication development, the majority of studies have primarily examined how eye gaze *accompanies* other communicative means (vocalizations, gestures). Thus, to date, it remains unclear what the communicative functions of infant eye gazing toward a communicative partner are.

### Communicative Functions

Because of our subjective experience and fragmentary knowledge of the world, communication is ambiguous, imprecise, and messy (Wittgenstein, 1962). The means that we use to communicate do not always convey a literal meaning; in fact, they are essentially equivocal. Linguists have examined this phenomenon by introducing the term “communicative act” – namely, the action (or set of actions) that a speaker accomplishes by producing a communicative bid. The Speech Act theory (Austin, 1962; Searle, 1969, 1975) supported the idea that utterances can have different levels of meaning at which actions are accomplished (Casillas and Hilbrink, 2020). It thus differentiated three types of acts: *perlocutionary acts* as the effect of having made an utterance; *illocutionary acts* as the effect on the listener which the speaker intended; and *locutionary* acts as the construction of propositions and uttering sounds.

Developmentally, there is a progression from perlocutionary to illocutionary and locutionary acts (Bates, 1979). Specifically, with their earliest communicative acts, it is unclear whether infants use them intentionally or not. Regardless, parents readily provide interpretations of the infant’s vocal and embodied activities as potentially meaningful communicative acts (perlocutionary acts; Bates, 1976). Around 10 months of age, infants begin to intentionally use a conventional signal in order to carry out some socially recognized functions (illocutionary acts). Through linguistic and/or embodied means (such as eye gaze, gesture), they start communicating the force or intent of a request, a command, a promise, or other social actions. When, by 12 months, children begin to use words with a referential value, they enter the stage of locutionary acts (Harding and Golinkoff, 1979).

In studies on early language development, researchers have primarily focused on perlocutionary and illocutionary acts. It is through the pioneering work of Elisabeth Bates on gesture communication that early communication development shifted from studying the *form* of acts infants use to communicate to the *function* of such communicative acts (Bates et al., 1975).

#### Perlocutionary Acts

In the first few months of life, infants produce a host of behaviors such as eye gaze, cries, smiles, and vocalizations that, at first, are not considered as being intentional but which lay the foundations for the development of communication and language (Adamson, 1996; Rochat, 2001). Infants produce such behaviors in the presence of another person, usually a parent, and a key feature of the response of parents is that they treat these behaviors as intentional and communicatively meaningful (see the concept of mind-mindedness offered by Meins, 1999). Thus, to a strident vocalization by a 6-month-old infant, a parent can respond, “You are not happy, are you?” Here, although it is unclear whether the infant intended to communicate dissatisfaction, the parent reacts as if he or she did. Bates et al. (1975) characterized such early behaviors as perlocutionary communication in that they often provide an effect on the parent.

#### Illocutionary Acts

Although her work spread across a large variety of topics, Bates’ main contributions related to the illocutionary force of infants’ early communication. Relying on Searle’s taxonomy of illocutionary acts (Searle, 1975; Searle and Vanderveken, 1985), Bates and her colleagues focused on performatives (Bates et al., 1979). Starting from the premise that infants and young children in the illocutionary stage (as early as 10 months of age) use non-verbal acts intentionally, two communicative functions were described: the proto-imperative and proto-declarative ones (Bates et al., 1975). The proto-imperative function relates to the child’s intentional use of the listener as an agent or tool in achieving some end. For example, a child might request a toy by looking fixedly at the parent’s face while extending his or her arm toward the toy. The proto-declarative function relates to the young child’s preverbal effort to direct the parent’s attention to some event or object in the world. An example is a child enthusiastic to share his or her interest with his or her parent by pointing to an interesting event, such as a clown riding a unicycle.

Beyond presenting perlocutionary and illocutionary acts in a separate way, it is fundamental to highlight that there is a clear developmental continuum from the first to the second. Specifically, it is primarily through the parental responses to infants’ earliest communicative acts (along with infants’ cognitive capacity for mean–end differentiation, linked to Piaget’s coordination of the secondary circular reactions substage of the sensorimotor stage; Dimitrova, 2013) that infants learn that their initial eye gaze, cries, smiles, vocalizations, and such can be actually used as a means to elicit a response from the parent (Bornstein et al., 1999).

### Communicative Functions of Eye Gaze

In humans, mutual gaze provides a foundation of communication and social interaction (Kendon, 1967, 1990; Kleinke, 1986; Csibra and Gergely, 2006; Rossano, 2013). Mutual gaze is an ostensive signal allowing establishment or re-establishment of a communicative link between two people (Csibra, 2010). In adult interaction, Patterson (1983)’s sequential model (1983) described the different functions of eye gaze including providing information, regulating interaction, exercising social control, etc.

In infancy, from the first weeks after birth, infants spend a significant amount of alert time in mutual gaze with a parent, and the large majority of early interactions are based on an infant’s interest in faces and mutual gaze (Volkmar, 1987). By 6 months of age, infants are equally likely to initiate an interaction both by gazing at their parent as well as by responding to gaze (Kaye and Fogel, 1980). These early interactions require mutual gaze and the interpersonal coordination of gaze. However, despite the fact that gaze is one of the fundamental resources organizing social interchanges and serves various functions in human interaction, there is a lack of studies examining when and how infants use eye gaze in order to communicate.

In the first 2 years of life, gaze serves various functions including engaging in social-communicative transactions and conveying non-verbal cues for social interaction (Volkmar and Mayes, 1990). First, infants are primarily absorbed by dyadic interactions with a parent. The functions of eye gaze during the first half of the first year are mainly related to emotional connectedness and sharing of emotional states. When they are engaged with a person, infants’ attention seems confined to the process of interaction itself. Thus, while young infants participate in finely tuned exchanges of affect, the system of communication is essentially expressive (Brazelton et al., 1974; Stem, 1974; Tronick et al., 1979). In the second half of the first year, infants’ interactions become gradually triadic as object-focused attention becomes embedded in social contexts (Werner and Kaplan, 1963; Trevarthen and Hubley, 1978; Bakeman and Adamson, 1984). Infants begin to switch their gaze back and forth between caregiver and object (Newson and Newson, 1975). Such gaze coordination indicates that infants are actively attempting to share attention to something external to the social interaction, establishing that object or event as the focus of joint concern (Murphy and Messer, 1977; Leung and Rheingold, 1981).

There are two notable exceptions of studies examining the function of eye gaze in early development. The first one includes the concept of social referencing, namely, situations in which an infant actively seeks – most commonly by gazing – an adult’s emotional expression to help interpret an ambiguous situation (see work on the Still Face procedure; Campos, 1983; Klinnert et al., 1983). The function of the infant’s eye gaze in social referencing might be characterized as social signaling, namely, the infant signals to the parent an *emotional distress* and seeks for cues in order to figure out how to act in an uncertain situation. Thus, while social referencing is a powerful communicative behavior during infancy, it is confined to the expression and exchange around (mostly negative) emotional states. The second exception of available studies on the function of gaze relates to infants’ capacity for gaze following (for an overview, see Flom et al., 2007). When infants follow the direction of attention of another person shifting to different features of the environment (i.e., attention-following) or when infants follow the direction of attention of another person toward a referent (i.e., referential gaze following; Scaife and Bruner, 1975; Butterworth and Jarrett, 1991), they gradually learn how another person’s attention can differ from their own. Although these abilities play a critical role in social learning, communication, and mental-state inferences (Argyle and Cook, 1976; Deák et al., 2008; Brooks and Meltzoff, 2014), they relate to an infant’s ability to *respond* to – but not to *initiate* – social interaction.

### Common Ground

How does a communicative partner access the intended meaning if it is not explicitly encoded in the communicative act? Since communication takes many forms and is often expressed indirectly, addressees must either rely on convention or infer what the speaker means from other available evidence. Theoretical accounts have pointed out that successful communication requires that partners share common ground about the referent in order to mutually access one another’s intention and thus determine the meaning of a given communicative act (e.g., Lewis, 1969; Schiffer, 1972; Stalnaker, 1978; Bruner, 1983; Tomasello, 1992, 2003; Clark, 1996). Common ground is a pool of experiences, knowledge, and meanings shared between communicative partners.

As Tomasello (2008) puts it, common ground is everything that we know and we know that we both know, also referred as third-order mentality. This aspect is particularly important for successful communication: producing a communicative act necessitates that one knows that the other will understand him or her based on the fact that the other knows that they both know what one means (e.g., I know what “cat” means, I know that you know what “cat” means, and I expect that you know that I know what “cat” means). The intention of a produced communicative act is thus being anchored in a common base of understanding shared between communicative partners. In social interaction, the already existing common ground is continuously being shaped and re-shaped by the participants’ verbal and embodied activities. Thus, a shared perceptual common ground (Clark, 1996) – including noticeable (visible, hearable, tangible) objects – emerges in real time and becomes mutually known to the participants through embodied and/or verbal acts of attention-sharing (Stukenbrock, 2018; Stukenbrock and Dao, 2019).

Concerning the nature of the content at stake, two general types of common ground about the communicative referent have been described (Clark, 1996; Tomasello, 2008). The first type is called perceptual co-presence, and it refers to the shared knowledge concerning elements from the immediate context of interaction. For example, when you are looking at a street performer and a person next to you says “It is amazing,” you would know what that person refers to and what he or she means relying on what you were both looking at (i.e., perceptual co-presence). The second type of common ground is called conceptual common ground and relates to a broader range of conventions such as norms and values that are shared in a given culture, society, or group. For example, if a person asks you if you know what time it is, you know that you are supposed to tell what time it is (and not merely answer “Yes, I know”) because you rely on conceptual common ground concerning interpersonal communication – in this example, formulation of polite requests.

Considering the very first communicative dynamics between an infant and a parent, it is crucial to understand how infants come to produce intentional communicative acts. Accordingly, a main question is how do infants come to share common ground with parents? Despite the importance of common ground in communication, little is known about the way infants construct and share meanings with their communicative partners. Common ground in infancy has been addressed empirically in a handful of experimental studies on perceptual co-presence in early communication (Ganea and Saylor, 2007; Moll et al., 2008; Liebal et al., 2009). Overall, these studies found that having previously shared a visual experience with a parent and a particular object helps infants as young as 14 months of age to disambiguate a parent’s pointing gestures in order to respond appropriately. Beyond perceptual co-presence, how does conceptual knowledge about the object shared between the young child and the parent shape the function of children’s early communicative acts?

### Framework for Studying Eye Gaze Functions

The existing evidence highlights that infants’ gaze toward a parent is a major meaningful behavior within social-communicative interactions. However, to date, it remains unclear what communicative functions eye gaze serves in the early stages of development. In this conceptual analysis, we provide a theoretical framework for the study of the functional aspects of eye gaze in early childhood. This theoretical framework relies on three intertwined premises: (1) the centrality of the object, (2) the importance of common ground on object use, and (3) the role of parental interpretations.

#### Centrality of the Object

An essential component of the developmental progression of infants’ abilities to communicate is the capacity to integrate an external object or event into the communicative exchange with another person (i.e., secondary subjectivity, Trevarthen and Hubley, 1978). It is precisely within such triadic interactions that infants begin to refer to the external world, primarily by referring to everyday objects from their immediate environment (Werner and Kaplan, 1963; Adamson, 1996), in order to convey requests (mainly regarding objects) or to obtain the parent’s attention (usually toward objects; Bates et al., 1979). Both of these performatives are first constructed on the plane of embodied action, namely, by manipulating objects rather than by formulating propositions. The centrality of the object has been emphasized in several processes of language acquisition and development such as the establishment of reference (e.g., Bruner, 1974), sharing joint attention (e.g., Adamson, 1996; Adamson and Dimitrova, 2014), the production of children’s first communicative gestures (i.e., pointing, e.g., Tomasello, 2008), as well as children’s first words (see Golinkoff et al., 1994). As genuine catalysts of communication between a parent and a child, objects are central to the early development of communication. In the theoretical framework that we provide, we argue that a meaningful analysis of the communicative functions of an infants’ eye gaze should consider the centrality of objects within triadic – parent–infant–object – early interactions (first premise).

#### Importance of Common Ground on Object Use

Objects have specific physical properties, such as size, shape, weight, color, softness, etc. Manufacturers specify the physical properties of objects, which provide for a certain amount of affordances (Gibson, 1979). However, the ways objects are used depend not only on their physical properties but on cultural conventions as well (Dimitrova, 2010, 2014). For example, while chopsticks afford a large number of uses (e.g., drumsticks, hairstyle accessory, gardening tools), there is a conventional type of use that is agreed upon and shared culturally. As adults, we master the conventional uses of everyday objects in our cultural environment (and we constantly adapt to the uses of novel objects). Infants, however, do not master such conventions even for simple objects from their immediate environment. They begin understanding that objects have specific uses within their interactions with their parents (Rodríguez and Moro, 1999; Moro and Rodríguez, 2005). For example, when an infant takes a spoon and bangs it continuously on a plastic plate, a parent typically picks it up from the child and shows how it should be used. Progressively, infants come to master conventional uses, and therefore, they begin sharing a type of common ground with others. Empirical evidence suggests that 7-month-old infants do not use objects according to the conventions of their use but, rather, perform undifferentiated actions such as banging, throwing, and mouthing objects (Fenson et al., 1976). Several months later, by 14 months of age, infants start appreciating the socio-cultural use of objects when they manipulate objects (Rodríguez and Moro, 1999; Moro and Rodríguez, 2005).

Previous studies suggest that sharing conceptual common ground (i.e., how to use objects in conventional ways) transforms – both quantitatively and qualitatively – the nature of the communicative interaction between infants and parents, allowing for important developmental progression. For example, Dimitrova and Moro (2013) have shown that when infants and parents share common ground on the conventional use of objects, parents increase both the amount and the complexity of their gestures. Specifically, when 8-month-old infants did not show any mastery of the conventional use of the toys with which they played during triadic parent–infant–object interactions, parents produced clear-cut gestures repeatedly while exaggerating their movement. Later on, by 14 months of age, when children showed that they were progressively learning the conventional use of toys, parents started producing more complex gestures with brief and fast movement, without repeating them.

Evidence suggests that when infants begin sharing common ground on the communicative referents with their parent, communication becomes functionally meaningful. In other words, when the infant and the parent share a type of common knowledge, they become progressively more skilled in producing and understanding each other’s communicative acts. Accordingly, in the theoretical framework that we provide, we argue that studying the communicative functions of infants’ eye gaze should include a consideration of the level of common ground shared between the parent and the infant (second premise).

#### Role of Parental Interpretations

In the study of the functions of young children’s first communicative gestures, Bates et al. (1975) showed a developmental progression between perlocutionary and locutionary acts. Namely, before young children’s ability to convey a clear communicative function (illocutionary acts), the study of the function of infants’ early communicative acts relies on the effect they trigger in the parent (perlocutionary acts). When parents are sensitive to an infant’s communicative signals, they interpret them accurately and respond to them appropriately. Importantly, parental interpretations and responses help young children discover the contingencies between their communicative productions and the effect they elicit on the parent, thus laying the foundations of the development of communication and language (Bornstein et al., 1999; Tamis-LeMonda et al., 2001).

There is empirical evidence suggesting that parents interpret infants’ early gestures based on shared knowledge about referents (Dimitrova et al., 2015). Studying the developmental progression of object use and of gesture production in 8- to 16-month-old infants, the authors found that when – by 14 months of age – infants started to show a mastery of the conventional use of objects, parents began to interpret the communicative function of their infants’ gestures referring to these objects as conveying a clear communicative function. The authors thus concluded that the level of common ground about the object shared between the parent and the infant was associated to the ability of parents to meaningfully interpret the function of their infant’s gestures.

Given the intrinsic difficulty of accessing the communicative intention of infants’ earliest communicative acts, analysis of the communicative function of infants’ eye gaze should consider the interpretations provided by parents (third premise).

## Discussion

In this conceptual paper, we provide a theoretical framework for the study of the communicative function of infants’ eye gaze. This theoretical framework relies on the following intertwined premises:

1. The centrality of the object: Beyond parent–child interactions in the first months of life when eye gaze primarily serves to establish connectedness and to share emotional states, studying the communicative function of infants’ eye gaze when infants become able to integrate an external object into their interaction with the parent requires a consideration of the object.
2. The importance of common ground on object use: Stemming from the first premise, examining the communicative function of infants’ eye gaze should take into consideration the level of common knowledge shared between the infant and the parent regarding the socio-cultural conventions of use of the referent of early communication, namely, the object.
3. The role of parental interpretations: When infants are at the early stages of their ability to convey communicative intentions, analysis of the function of infants’ eye gaze should consider the parental interpretations of the function of the infant’s gaze.

Based on previous studies on the importance of common ground on objects used for early communication (Dimitrova and Moro, 2013; Dimitrova et al., 2015), we argue that when infants and parents start sharing common ground on the conventional use of the objects/toys, parents become more likely to provide accurate interpretations of the function of their infants’ eye gaze behavior. As described in early development, children’s early communicative acts serve two primary functions: requesting something from the parent (i.e., proto-imperative) and sharing attention and interest with the parent (i.e., proto-declarative; see Bates et al., 1975). In order to study what young children mean with their eye gaze – and more specifically, how parents interpret the communicative function of their infants’ eye gaze – we argue that it is crucial to examine the knowledge parents and children share about the objects that they refer to in their communicative interactions. Importantly, the variability in children’s mastery of the conventional use of objects is likely related to the variability in communicative functions that parents attribute to their children’s eye gaze. Namely, when children are in the process of acquiring the conventional use of objects (i.e., proto-conventional use), the uncertainty and difficulty that they display in their object use might cue parents to interpret children’s eye gaze, either as a request for help or assistance (i.e., proto-imperative function) or as an invitation to share attention and interest (i.e., proto-declarative function). However, when children master the conventional use of objects, the level of shared common ground should allow parents to interpret children’s eye gaze as a way to establish joint attention and/or to seek validation or encouragement.

The theoretical premises provided in this paper are a first step toward the study of the functional aspects of eye gaze in early childhood. In order to examine the scientific soundness of this theoretical framework, empirical data are required. We recommend that analysis of the function of infants’ eye gaze rely on naturalistic observations of parent–infant interactions with objects, where both the infants’ level of mastery of the socio-cultural uses of objects (i.e., common ground) and the nature of parental responses to infant eye gaze are assessed. It is important to highlight that early intentional communication succeeds generally when different communicative means are solicited. Despite the fact that we focus on eye gaze, analysis of the functional aspects of infants’ communication should include the multimodal means – such as vocalizations, gestures, and body postures – that infants mobilize together with eye gaze when communicating.

To our knowledge, this conceptual analysis is the first effort to provide a theoretical framework for the study of the functional aspects of eye gaze in early childhood. Indeed, despite the fact that eye gaze is a crucial component of interaction and communication (Kendon, 1967, 1990; Kleinke, 1986; Csibra and Gergely, 2006; Rossano, 2013), the communicative functions of child eye gaze are understudied. While pragmatic accounts of children’s early communicative development are widespread in the literature, questions related to the underlying processes driving the development of children’s abilities to convey different communicative functions remain understudied. Admittedly, there are various processes contributing to children’s ability to communicate their intentions. Nonetheless, relying on the importance of the shared common knowledge about the main focus of early parent–child communication – namely, the object – allows for a framework for understanding how infants develop their abilities to communicate intentionally.

In this conceptual analysis, we hypothesize that common ground shared between the parent and the infant about the use of an object would allow parents to interpret the function of their infants’ eye gaze as communicatively meaningful. We argue that the knowledge on the communicative referent shared between the infant and the parent shapes the course of communicative behavior, constitutes and reflects the interactive function of gaze, and cues parents into tailoring their communicative response according to the infant’s developmental needs, which in turns feeds back into the infant’s social and communicative development.

## Ethics Statement

Ethical review and approval were not required for the study on human participants in accordance with the local legislation and institutional requirements. The patients/participants (and their guardians in the case of participants under the age of 16) provided their written informed consent to participate in this study.

## Author ContRibutions

ND conceptualized the theoretical framework and wrote the manuscript.

## Conflict of Interest

The author declares that the research was conducted in the absence of any commercial or financial relationships that could be construed as a potential conflict of interest.
